# Late-course accelerated Hyperfractionation vs. Conventional Fraction Radiotherapy under precise technology plus Concurrent Chemotherapy for Esophageal Squamous Cell Carcinoma: comparison of efficacy and side effects

**DOI:** 10.7150/jca.41012

**Published:** 2020-03-04

**Authors:** Hongtao Luo, Shihong Wei, Xiaohu Wang, Ruifeng Liu, Qiuning Zhang, Zhen Yang, Zheng Li, Xiyi Wei, Yuexiao Qi, Lijun Xu

**Affiliations:** 1 The First Clinical Medical College of Lanzhou University, Lanzhou 730000, China.; 2 Gansu Provincial Cancer Hospital, Lanzhou 730050, China.; 3 The Basic Medical College of Lanzhou University, Lanzhou 730000, China.; 4 Lanzhou Heavy Ion Hospital, Lanzhou 730000, China.; 5 Institute of Modern Physics, Chinese Academy of Sciences, Lanzhou 730000, China.

**Keywords:** Esophageal cancer, late course accelerated hyperfractionation, Intensity-modulated radiotherapy, Image-guided radiotherapy, concurrent chemoradiotherapy

## Abstract

**Background**: The accelerated reproliferation of esophageal squamous cell carcinoma (ESCC) after radiation contributes to conventional fraction radiotherapy (CFRT) failure. Late course accelerated hyperfractionated radiotherapy (LCAHFRT) can improve the long-term survival of esophageal cancer patients in China but is associated with a high rate of side effects due to the large exposure field of two-dimensional treatment and drug toxicity. Intensity-modulated radiotherapy (IMRT) can increase the tumor dose while decreasing the normal tissue dose. Therefore, we compared the outcomes and side effects of LCAHFIMRT plus concurrent chemotherapy (CT) and CFIMRT plus CT for ESCC.

**Methods and Materials**: Between 2013 and 2016, 114 eligible patients with ESCC were recruited and randomly assigned to receive LCAHFIMRT+CT (58 patients) or CFIMRT+CT (56 patients) by a linear accelerator (6-MV X-ray) under image guidance. Two cycles of CT with cisplatin and docetaxel were also administered.

**Results**: The complete response (CR) rates were 79.3% and 61.8% in the LCAHFIMRT+CT and CFIMRT+CT groups, respectively (P=0.041). The median duration of local control times was 31.0±1.9 months for the LCAHFIMRT+CT group and 24.0±3.3 months for the CFIMRT+CT groups,and the 1-, 2-, and 3-year local control rates were 86.2%, 63.8%, and 41.4% and 85.7%, 51.8%, and 32.1% for the LCAHFIMRT+CT and CFIMRT+CT groups (P=0.240), respectively. The median survival times were 34.0±1.1 months for the LCAHFIMRT+CT group and 28.0.0±3.7 months for the CFIMRT groups,and the 1-, 2-, and 3-year survival rates were 87.9%, 74.1%, and 44.8% and 87.5%, 60.7%, and 39.3% for the LCAHFIMRT+CT and CFIMRT+CT groups, respectively (P=0.405). The incidence of side effects was not significantly different between the two groups. Local recurrence and uncontrolled disease resulted in more deaths in the CFIMRT+CT group than in the LCAHFIMRT+CT group (58.9% vs. 39.7%) (P=0.040).

**Conclusion**: For ESCC patients, LCAHFRT delivered by image-guided intensity-modulated techniques Plus Concurrent Chemotherapy with cisplatin and docetaxel keeps safety and high CR rate, as well as local control and long-term survival rates.

## Introduction

Esophageal cancer (EC) is the 7th most common malignancy and the 6^th^ most common cause of cancer death around the world; approximately 572,000 new cases of EC and 509,000 deaths related to EC occurred in 2018 1. Among these cases, approximately 80% of the cases of morbidity and mortality occurred in developing countries 2. In China, the incidence of EC and deaths related to EC account for 50% of all cases globally around the world and 60% of those in developing countries. EC is still the main burden of disease for local residents, especially in the rural areas of the Midwest which are considered high-incidence areas 3, 4.

Although surgery is an effective treatment for EC, most patients tend to be at an advanced stage at the time of diagnosis 5,6. Thus, radiotherapy (RT) plays a well-defined role in the management of patients with inoperable locally advanced EC 7. However, experimental studies have shown that the accelerated re-proliferation of tumor cells after radiation is an important reason for failed conventional fraction radiotherapy (CFRT) 8. Several clinical trials verified that nonconventional fraction RT, especially late course accelerated hyperfractionated radiotherapy (LCAHFRT), can improve the long-term survival of patients with esophageal cancer 9-11. In 1999, Shi XH et al. reported that compared with CFRT, LCAHFRT improved the 5-year survival rate from 26% to 33% 10. Zhao KL et al. used LCAHFRT with concurrent chemotherapy (CT) for patients with ESCC and found that this method was comparable to LCAFRT alone; patients who received concurrent LCAFRT and CT exhibited better survival with a 5-year survival rate of 40% and a median survival time of 30.8 months 11. However, a large number of side effects were reported in these studies due to the large exposure field of two-dimensional treatment and drug toxicity.

Currently, advanced RT techniques such as intensity-modulated radiotherapy (IMRT) and image-guided radiotherapy (IGRT) have progressively changed the practice of treating EC by precisely irradiating the target while minimizing the risk of damaging normal tissues 12-15. Image-guided intensity-modulated radiotherapy (IG-IMRT) becomes an attractive modality largely owing to the geometrical uncertainties such as respiratory motion and day-to-day position variability of the tumor 16.

The Radiation Therapy Oncology Group (RTOG) phase Ⅲ intergroup trials RTOG 85-01 and 94-05 showed that RT plus concurrent 5-fluorouracil (5-FU) and cisplatin (DF) is the standard CT regimen for patients with EC 17, 18. Novel CT drugs applied in clinical application have significantly improved the curative effects of CT while alleviating the side effects. Docetaxel is a clinically well-established anti-mitotic CT medication that affects dynamic microtubule assembly and disassembly 19. Yang HX et al. (2015) reported that the use of neoadjuvant CT agents docetaxel and nedaplatin may provide excellent outcomes for down-staging disease and R0 resection and the patients with EC in their study had a 2-year survival rate of 71.1% 20.

However, virtually all seminal clinical trials for LCAFRT in EC have utilized two-dimensional radiotherapy (2DRT) or three-dimensional conformal radiotherapy (3DCRT) 9-11,21. There was a lack of supporting data for LCAFRT in EC under IG-IMRT. Therefore, LCAHFRT delivered by image-guided intensity-modulated techniques in combination with new CT regimens with cisplatin and docetaxel should be further investigated to assess the efficacy and side effects of this method in ESCC patients.

## Materials and Methods

### Eligibility criteria

The eligibility criteria were as follows: (1) histologically confirmed squamous cell carcinoma of the esophagus; (2) age 18~75 years old with Karnofsky performance status (KPS) scores ≥60; (3) stage I to IV disease according to the 2017 (version 8.0) American Joint Committee on Cancer staging system, with the exception of T4b and M1 disease; (4) life expectancy of at least 6 months; (5) normal baseline laboratory tests (white blood cell count ≥3.5×10^9^/L, platelet count ≥125×10^9^/L, and hemoglobin ≥115 g/L); (6) normal renal function (serum creatinine<106 µmol/L, blood urea<8.63 mmol/L); (7) normal liver function (total serum bilirubin≤20.5 µmol/L and aspartate transaminase and alanine transaminase levels lower than double the upper normal limit); and (8) adequate pulmonary function. The exclusion criteria were as follows: (1) previous treatment with any other therapy, i.e., surgery, CT, or targeted drugs; (2) esophageal perforation or deep ulceration; (3) esophageal bleeding; (4) complete esophageal obstruction; (5) distant metastases; and (6) concomitant serious illness such as uncontrolled angina pectoris, heart failure, interstitial pneumonia, or infection or other diseases contraindicating CT or RT.

### Pretreatment evaluations

The pretreatment evaluation included obtaining a medical history and performing a physical examination, routine blood and biochemistry test, electrocardiogram (ECG) measurements, esophageal barium examination, contrast-enhanced neck and chest computed tomography and ultrasonography of the heart and abdomen, radionuclide bone scan, and magnetic resonance imaging (MRI); Positron emission tomography-computed tomography (PET-CT) were used when clinically needed. This study was approved by the Ethics Committee of Gansu Tumor Hospital, Lanzhou, China. Informed consent was provided by the patient or their legal representatives.

### Study design

The patients were randomized into two groups by a random number table. In the study group, the patients received LCAHFIMRT+CT, and in the control group, the patients received CFIMRT+CT.

### Radiation and target definition

Radiation for IMRT was carried out by a linear accelerator (6-MV X-ray). Each patient underwent CT imaging with an intravenous contrast agent for treatment planning. Then, the images were transferred to a radiotherapy planning system (Eclipse). The target area was delineated by two associate chief physicians. The gross tumor volume was defined as the macroscopic primary tumor (GTV) and regional lymph node metastases (GTVnd) based on the endoscopy, endoscopic ultrasonography, barium esophagography and chest CT scans. The clinical target volume (CTV) was defined as the GTV/GTVnd plus a 0.5-1 cm radical margin and 3-4 cm cranio-caudal margin. The supraclavicular nodes were included as upper esophageal lesions, and the celiac nodes were included as distal esophageal lesions. The planning target volume (PTV) consisted of the CTV plus a 0.5-1 cm margin for daily setup error and organ motion.

### Fractionated dose schemes

In the present study, all patients received simultaneous integrated boost IMRT (SIB-IMRT) under imaging guided. In the LCAHFIMRT group, the prescribed dose was divided into a two-phase irradiation schedule. The first phase was conventional fractionated irradiation with 44 Gy/2.2 Gy/20 fractions to the GTV and GTVnd and 36 Gy/1.8 Gy/20 fractions to the CTV with five fractions per week; the second irradiation phase was the accelerated hyperfractionated session. The dose was delivered twice per day with a minimum interval of 6 hours for 10 fractions per week with 18 Gy/1.5 Gy/12 fractions to the GTV and GTVnd and 13.2 Gy/1.1 Gy/12 fractions to the CTV. The two-phase treatment plan was merged together by the MIM system. The total dose of the two-phase irradiation regimen was 62 Gy for the GTV and GTVnd and 49.2 Gy to the CTV in 32 fractions. In the CFIMRT group, the prescribed dose was 2.2 Gy per fraction for five fractions per week with 61.6 Gy to the GTV and GTVnd and 1.8 Gy per fraction with 50.4 Gy for CTV in 28 fractions for five times a week. For the normal tissue, the percentage of the whole lung volume that received more than 20 Gy irradiation (V_20_) was ≤28%, and the percentage that received more than 30 Gy (V_30_) was ≤20%, the cardiac mean dose (Dmean) was <30 Gy, and the maximum dose to the medulla spinal was <45 Gy.

### IGRT

Daily cone beam computed tomography(CBCT) scans (Synergy 4.5, Elekta Ltd, Sweden) were acquired and bony anatomy registration was used for online setup error correction if the error <1cm, otherwise, setup was required again when the error ≥1cm.

### Chemotherapy

All patients received two cycles of concurrent CT with a regimen consisting of cisplatin and docetaxel; cisplatin was provided at 25 mg/m^2^/day i.v. from days 1-3, and docetaxel was provided at 60 mg/m^2^/day i.v. on day 1. The first cycle began on the first day of RT, while the second cycle of CT began on the twenty-eighth day of RT if the patients did not exhibit side effects that exceeded grade 2 or if the investigator decided that the patient should not receive the second cycle of CT.

### Efficacy evaluation

The treatment effect was evaluated according to the Response Evaluation Criteria in Solid Tumors (RECIST, version 1.1) after three months of CT and was classified into complete response (CR), partial response (PR), stable disease (SD), and progressive disease (PD) 23. Long-term effects were evaluated by the median local control time, median survival time, 1-, 2- and 3-year local control rates and 1-, 2- and 3-year survival rates.

### Side effect criteria

The acute and long-term side effects were evaluated by the American Radiation Therapy Oncology Group (RTOG) criteria severity scales (http://www.rtog.org./). Acute side effects appeared between the beginnings of treatment to three months after completing treatment. Long-term side effects were recorded at each patient follow-up visit.

### Statistical analysis

The data were analyzed using SPSS 21.0 software. The constituent ratio was analyzed by the χ^2^ test, and the measurement data were analyzed by the nonparametric test; the total survival rate and local control rate were calculated by the Kaplan-Meier method, and the significance of the differences was examined by the log-rank method. A P value less than 0.05 indicated statistical significance.

## Results

### Patient characteristics

Between January 2013 and June 2016, 114 patients with esophageal squamous cell carcinoma (ESCC) were randomized to the study. The pretreatment characteristics of the 114 eligible and assessable patients are listed in Table 1. The two randomized groups were well balanced regarding sex, age, tumor location, primary tumor length, tumor stage, nodal stage, clinical stage, histological grade, KPS score and weight loss before treatment.

### Treatment and follow-up

In the LCAHFIMRT+CT group, the highest radiation dose to the GTV or GTVnd was 62 Gy, and the minimum dose was 52 Gy; the corresponding values were 61.6 Gy and 55 Gy in the CFIMRT+CT group. All patients in both groups completed two cycles of CT. The patients were followed up every three months for two years, then every six months for two years, and then every year. The last follow-up occurred in June 2019. The follow-up periods were 36-58 (median 45) months, and the follow-up rate was 100%.

### Short-term curative effect

The CR, PR, SD and PD were 79.3%, 10.3%, 6.9% and 3.4% in the LCAHFIMRT+CT group and 61.8%, 23.2%, 10.7% and 5.4% in the CFIMRT+CT group, respectively; the CR was significantly different between the LCAHFIMRT+CT group and CFIMRT+CT group (P=0.041), but the PR, SD and PD were not significantly different between the two groups (P=0.065, 0.417 and 0.619, respectively), as shown in Table 2.

### Local control rate and survival rate

**Figure 1:**
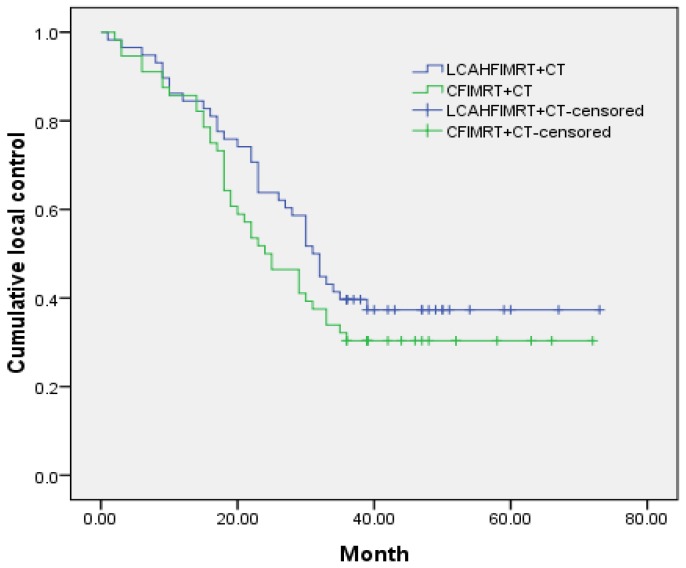
** Comparison of local control rates.** The 1-, 2-, and 3-year-local control rate curves (data shown in months) are compared in the LCAHFIMRT+CT group and the CFIMRT+CT group of ESCC.

**Figure 2:**
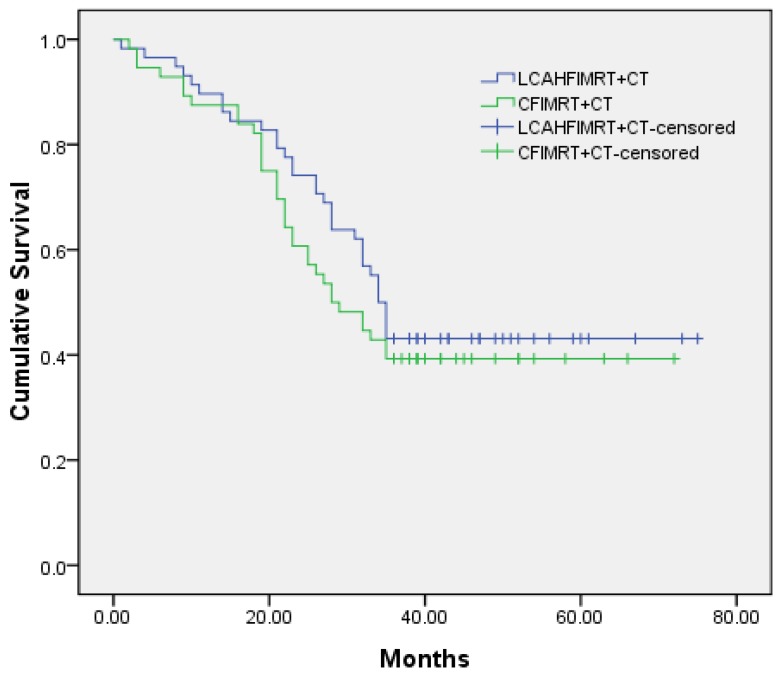
** Comparison of survival rates.** The 1-, 2-, and 3-year survival rate curves (data shown in months) are compared in the LCAHFIMRT+CT group and the CFIMRT+CT group of ESCC.

The median local control times were 31.0±1.9 months (95% CI 27.3-34.7) in the LCAHFIMRT+CT group and 24.0±3.3 months (95% CI 17.5-30.5) in the CFIMRT+CT group, respectively; the 1-, 2-, and 3-year local control rates were 86.2%, 63.8%, and 41.4% in the LCAHFIMRT+CT group and 85.7%, 51.8%, and 32.1% in the CFIMRT+CT group, respectively. The differences between the two groups were not significant (Fig. 1, χ^2^ =1.383, P=0.240). The median survival times were 34.0±1.1 months (95% CI 31.9-36.1) in the LCAHFIMRT+CT group and 28.0±3.7 months (95% CI 20.7-35.3) in the CFIMRT group, respectively. The 1-, 2-, and 3-year survival rates were 87.9%, 74.1%, and 44.8% in the LCAHFIMRT+CT group and 87.5%, 60.7%, and 39.3% in the CFIMRT+CT groups, respectively. The differences between the two groups were not significant (Fig. 2; χ^2^= 0.693, P=0.405).

### Side effects of treatment

The incidence of acute and late side effects in the LCAHFIMRT+CT group was slightly higher than the CFIMRT+CT group. Because nutritional supplementation and symptomatic treatment were administered to patients throughout the study period, the main side effects were below grade 3. In terms of severe side effects: 2 cases of esophageal perforation and 2 cases of pulmonary fibrosis were noted in the LCAHFIMRT+CT group, whilst 1 case of esophageal perforation and 3 cases of pulmonary fibrosis were noted in the CFIMRT+CT group, which was not found to be a significant difference (P>0.05) (Table 3).

### Cause of death

At the last follow-up, A total of 43 patients died in the LCAHFIMRT+CT group, due to the following: local recurrence or uncontrolled disease 39.7% (23/58); distant metastasis 25.9% (15/58); and therapeutic side effects 13.8% (8/58). A total of 45 patients died in the CFIMRT+CT group due to the following: local recurrence or uncontrolled disease 58.9% (33/56); distant metastasis 39.3% (22/56); and therapeutic side effects 12.5% (7/56). The incidence of local recurrence or uncontrolled disease was significantly different between the two groups (P=0.040), but the incidences of distant metastasis and therapeutic side effects were not (P=0.126 and 0.838, respectively). The data are shown in Table 4.

## Discussion

Radiation plays a crucial role in the treatment of EC; however, the 5-year survival rate of EC patients treated with RT alone is 23.31±10.21% due to the high rates of failed local recurrence and uncontrolled disease 7. The accelerated reproliferation of tumor cells after RT is another important reason for RT failure 8. In a 2DRT era, Zhao KL et al. reported that LCAHFRT together with CT using the DF regimen led to a median survival of 30.8 months and 1-, 3-, and 5-year survival rates of 67%, 44%, and 40%, respectively 11. Wang JH et al. showed that the 1-, 2-, and 3-year local control rates and survival rates of 3-dimensional late course accelerated hyperfractionated conformal radiotherapy were 81.3%, 62.5%, and 50% and 79.2%, 56.3%, and 43.8%, respectively^21^. Thus, high-dose radiation did not lead to improved curative effects for EC patients. In the present study, all patients received image-guided conformal IMRT, which utilizes computer-controlled linear accelerators to deliver precise radiation doses to the GTV and GTVnd as well as CT-based image guidance and repositioning prior to RT. Li CC et al. 23 showed that concurrent chemoradiation (CCRT) coupled with IGRT is associated with better overall survival compared with CCRT without IGRT in patients with nonoperable localized ESCC.

The phase III RTOG intergroup trials 85-01 and 94-05 established the value of CCRT as the standard therapy for patients with EC patients 17,18, and the DF regimen was recommended as the first-line CT for EC 24. However, the DF regimen has limited efficacy and application due to the mucosal response to 5-FU. Compared to the DF regimen, docetaxel combined with cisplatin had synergistic effects at lower concentrations and promoted apoptosis but did not increase the side effects of CT 25. Sasaki K et al. used neoadjuvant chemoradiotherapy with docetaxel/cisplatin/5-FU (DCF) in 30 patients with ESCC. The 3-year overall survival rate was 62.2%, and the 3-year pathologic complete response (pCR) rate was 84% 26. Combination chemotherapy using DCF has shown promising efficacy for patients with ESCC in adjuvant and salvage settings 27, 28. In our study, we used LCAHFIMRT plus CT in view of the high dose of radiotherapy for patients and a two-drug regimen with docetaxel and cisplatin (DC) for concurrent chemotherapy. The results showed that the CR rate was 79.3% in the LCAHFIMRT+CT group, which was higher than that in the CFIMRT+CT group (61.8%). Compared to CFIMRT+CT, LCAHFIMRT+CT prolonged the median local control time and the median survival time of patients. The 1-, 2-, and 3-year local control rates and survival rates were 86.2%, 63.8%, and 41.4% and 87.9%, 74.1%, and 44.8%, respectively, in the LCAHFIMRT+CT group, which were higher than those in the CFIMRT+CT group as well as those reported in previous studies. The acute and late side effects that occurred were mainly grade I and II in both the LCAHFIMRT+CT group and the CFIMRT+CT group, possibly because IMRT reduces the radiation dose to the surrounding normal tissue and because not all patients received adjuvant CT after CCRT in our study. However, we observed 6 cases and 5 cases of fatal serious side effects in the esophagus and lung in the LCAHFIMRT+CT group and the CFIMRT+CT group, respectively. The main causes of death were local recurrence or uncontrolled disease in both groups.

Overall, our results revealed that image-guided LCAHFIMRT combined with new CT regimens with cisplatin and docetaxel, which had a better short-term efficacy and a slightly higher local control rates and long-term survival rates than CFIMRT for ESCC. The side effects in the LCAHFIMRT+CT group were similar to that in the CFIMRT group owing to use the IG-IMRT and new chemotherapy regimens. However, improving the local control rate and reducing the long-term side effects of CCRT are still difficult problems to solve in clinical practice.

## Figures and Tables

**Table 1:** Clinical characteristics of esophageal cancer patients n (%)

Feature	LCAHFIMRT+CT group 58 case	CFIMRT+CT group 56 case	χ^2^	P
**Gender**			0.386	0.535
Male	34(58.6%)	36(64.3%)		
Female	24(41.4%)	20(35.7%)		
**Age (years)**			1.723	0.423
≤50	15(25.9%)	12(21.4%)		
50~70	24(41.4%)	30(53.6%)		
≥70	19(32.8%)	14(25.0%)		
**Tumor location**			1.459	0.482
Upper	22(37.9%)	19(33.9%)		
Middle	17(29.3%)	22(39.3%)		
lower	19(32.8%)	15(26.8%)		
**Primary tumor length**			0.860	0.354
<5cm	17(29.3%)	21(37.5%)		
≥5cm~<10	41(70.7%)	35(62.5%)		
**Tumor stage**			2.277	0.517
T1	5(8.6%)	4(7.1%)		
T2	22(37.9%)	28(50.0%)		
T3	29(50.0%)	21(37.5%)		
T4a	2(3.4%)	3(5.4%)		
**Nodal stage**			1.245	0.537
N0	38(65.5%)	37(66.1%)		
N1	6(10.3%)	9(16.1%)		
N2	14(24.1%)	10(17.9%)		
**Clinical stage**			0.307	0.998
I	4(6.9%)	4(7.1%)		
IIB	17(29.3%)	16(28.6%)		
IIA	16(27.6%)	16(28.6%)		
IIIA	9(15.5%)	8(14.3%)		
IIIB	10(17.2%)	9(16.1%)		
IVA	2(3.4%)	3(5.4%)		
**Histological grade**			3.181	0.204
well	22(37.9%)	19(33.9%)		
moderately	32(55.2%)	27(48.2%)		
poorly	4(6.9%)	10(17.9%)		
**KPS**			1.417	0.234
≥60~≤770	26(44.8%)	19(33.9%)		
≥80	32(55.2%)	37(66.1%)		
**Weight loss before therapy**			1.698	0.193
<10%	39(67.2%)	31(55.4%)		
≥10%	19(32.8%)	25(44.6%)		

*LCAHFIMRT: late course accelerated hyperfractionated intensity modulated radiotherapy; CFIMRT: conventional fraction intensity modulated radiotherapy; CT: chemotherapy.

**Table 2:** Comparison of short-term curative effects n (%)

Feature	LCAHFIMRT+CTgroup 58 case	CFIMRT+CTgroup 56 case	χ^2^	P
CR	46(79.3%)	34(61.8%)	4.178	0.041
PR	6(10.3%)	13(23.2%)	3.398	0.065
SD	4(6.9%)	6(10.7%)	0.519	0.471
PD	2(3.4%)	3(5.4%)	0.248	0.619

*LCAHFIMRT: Late course accelerated hyperfractionated intensity modulated radiotherapy; CFIMRT: conventional fraction intensity modulated radiotherapy; CT: chemotherapy; CR: complete response; PR: partial response; SD: stable disease; PD: progressive disease.

**Table 3:** Comparison of treatment side effects

Reasons of death	Group		χ^2^	P
	LCAHFIMRT+CT	CFIMRT+CT		
Number of cases	43	45		
Local recurrence or Uncontrolled disease	23(39.7%)	33(58.9%)	4.235	0.040
Distant metastasis	15(25.9%)	22(39.3%)	2.342	0.126
Therapeutic side effects	8(13.8%)	7(12.5%)	0.042	0.838

*LCAHFIMRT: Late course accelerated hyperfractionated intensity modulated radiotherapy; CFIMRT: conventional fraction intensity modulated radiotherapy; CT: chemotherapy.

**Table 4:** Comparison of the causes of death, n (%)

Reasons of death	Group		χ^2^	P
	LCAHFIMRT+CT	CFIMRT+CT		
Number of cases	43	45		
local recurrence or uncontrolled disease	23(39.7%)	33(58.9%)	4.235	0.040
Distant metastasis	15(25.9%)	22(39.3%)	2.342	0.126
Therapeutic side effects	8(13.8%)	7(12.5%)	0.042	0.838

*LCAHFIMRT: Late course accelerated hyperfractionated intensity modulated radiotherapy; CFIMRT: conventional fraction intensity modulated radiotherapy; CT: chemotherapy.
